# The LIM homeodomain transcription factors Lhx6 and Lhx7 are key regulators of mammalian dentition

**DOI:** 10.1016/j.ydbio.2009.07.001

**Published:** 2009-09-15

**Authors:** Myrto Denaxa, Paul T. Sharpe, Vassilis Pachnis

**Affiliations:** a Division of Molecular Neurobiology, MRC National Institute for Medical Research, The Ridgeway, Mill Hill, London NW7 1AA, UK; b Department of Craniofacial Development, King's College, Guy's Hospital, London SE1 9RT, UK

**Keywords:** LIM homeodomain, *Lhx6*, *Lhx7*, Odontogenesis, Molars, Incisors

## Abstract

Genes encoding LIM homeodomain transcription factors are implicated in cell type specification and differentiation during embryogenesis. Two closely related members of this family, *Lhx6* and *Lhx7*, are expressed in the ectomesenchyme of the maxillary and mandibular processes and have been suggested to control patterning of the first branchial arch (BA1) and odontogenesis. However, mice homozygous for single mutations either have no cranial defects (*Lhx6*) or show only cleft palate (*Lhx7*). To reveal the potential redundant activities of *Lhx6* and *Lhx7* in cranial morphogenesis, we generated mice with all combinations of wild-type and mutant alleles. Double homozygous mice have characteristic defects of the cranial skeleton and die shortly after birth, most likely because of cleft palate. In addition, Lhx6/7 deficient embryos lack molar teeth. The absence of molars in double mutants is not due to patterning defects of BA1 but results from failure of specification of the molar mesenchyme. Despite molar agenesis, Lhx6/7-deficient animals have normal incisors which, in the maxilla, are flanked by a supernumerary pair of incisor-like teeth. Our experiments demonstrate that the redundant activities of the LIM homeodomain proteins Lhx6 and Lhx7 are critical for craniofacial development and patterning of mammalian dentition.

## Introduction

One of the best-studied models of mammalian craniofacial organogenesis is tooth development. As with many other organs, mammalian tooth development begins early in embryogenesis by a series of reciprocal signalling interactions between the stomodeal ectoderm and the underlying neural crest-derived ectomesenchyme (Tucker and Sharpe, 2004). The first sign of mammalian tooth development is the thickening of the oral epithelium at the sites of the future teeth. Subsequently, the epithelium invaginates into the underlying mesenchyme, which responds by condensation, forming a tooth bud. The epithelium then folds further into the condensing mesenchyme and surrounds it to form initially the “cap” and then the “bell” stage tooth germ. Expression pattern analysis, manipulation of organ cultures and generation of knockout mice for several secreted signalling molecules and transcription factors have increased our understanding for several of these processes (reviewed in Peters and Balling, 1999; Jernvall and Thesleff, 2000; Cobourne and Sharpe, 2003, Tucker and Sharpe, 2004; Chai and Maxson, 2006).

Normal dental patterning in mice depends on the accurate choice of the position, subtype and number of future teeth. Prior to the initiation of tooth development the neural crest-derived mesenchyme of the first branchial arch is subdivided into oral and aboral components, which develop teeth and the skeletal elements of the jaw, respectively. The proximal part of the maxillary and mandibular processes gives rise to molars while the distal domain forms incisors. Recent evidence suggests that the regionally restricted, combinatorial expression of homeobox-transcription factors, such as members of the Dlx, Msx, Gsc, Brx and Lhx families, is responsible for generating the early polarity of the mandibular arch (Thomas et al., 1997; Tucker et al., 1998, 1999). On the other hand, the activation level of specific signalling pathways, such as those of ectodysplasin and Fgf, is thought to have an important role in defining tooth number (Mikkola and Thesleff, 2003; Mustonen et al., 2003; Tucker et al., 2004; Courtney et al., 2005; Klein et al., 2006). Despite significant progress in our understanding of the patterning of mouse dentition, studies of mouse mutants with characteristic changes in the relative number of tooth subtypes promise to unravel some of the developmental complexities of mammalian odontogenesis.

LIM/homeodomain genes encode transcription factors, which are characterized by the association of two LIM domains with a homeodomain. A large body of literature supports the notion that LIM domain functions as a protein–protein interaction motif, which regulates the binding of the homeodomain to the DNA (Schmeichel and Beckerle, 1994; Arber and Caroni, 1996). Several members of this family have been implicated in regulating specific aspects of patterning and differentiation in several tissues, including the nervous system and the developing limbs in both invertebrate and vertebrate organisms (Bach, 2000; Hobert and Westphal, 2000).

*Lhx6* and *Lhx7* (also termed *L3* or *Lhx8*) (Grigoriou et al., 1998; Matsumoto et al., 1996a,b, Kitanaka et al., 1998) belong to the most divergent subgroup of LIM homeodomain encoding genes (Hobert and Westphal, 2000), which also includes the *Drosophila arrowhead* (Curtiss and Heilig, 1995) and the *C. elegans lim-4* (Sagasti et al., 1999). During development, both genes are expressed in overlapping domains of the forebrain, the oral ectomesenchyme of the maxillary and mandibular processes of the first branchial arch, and the palatal shelves (Matsumoto et al., 1996a,b; Grigoriou et al., 1998). In addition, the chick orthologues of *Lhx6* and *Lhx7* are expressed in the mesenchyme of the lateral globular projections of the medial nasal process (Washbourne and Cox, 2006). *In vitro* studies have suggested that Fgf8 acts as a strong inducer of *Lhx6* and *Lhx7* (Grigoriou et al., 1998), which are thought to be part of the mechanisms that restrict expression of *Gsc* in the caudal mesenchyme, thus establishing the oral–aboral polarity in the mandibular arch (Tucker et al., 1999). *Lhx6* and *Lhx7* expression persists at later developmental stages in the mesenchyme of individual teeth, suggesting a possible role for these genes in tooth formation (Grigoriou et al., 1998; Shibaguchi et al., 2003). In support of this idea, tooth germ explants from E12.5 mice (E = embryonic day) treated with antisense-oligodeoxynucleotides against *Lhx7*, showed a marked decrease in the number of mesenchymal cells and absence of tooth germ formation (Shibaguchi et al., 2003). Despite these studies, mice deficient for *Lhx6* have no obvious craniofacial defects, while 70% of the *Lhx7* mutants show only palatal defects (Zhao et al., 1999; our observations), suggesting redundant activities of these genes in craniofacial development and odontogenesis.

To study the potential genetic interactions between *Lhx6* and *Lhx7* in craniofacial development, we generated mice with all combinations of wild-type and null alleles at these loci. Our experiments show that the combined deletion of *Lhx6* and *Lhx7* does not alter the molecular and anatomical asymmetry of the first branchial arch along the oral–aboral axis, but results in loss of all molar teeth. The loss of molars is due to failure of the mesenchyme of the molar domain to be specified as odontogenic. We also demonstrate that in contrast to molars, the combined deletion of *Lhx6* and *Lhx7* does not affect the development of incisors, which in the maxilla are flanked by an additional pair of incisor-like teeth embedded in the most distal part of the diastema domain. We propose that Lhx6 and Lhx7 LIM homeodomain proteins are key regulators of mammalian odontogenesis, which control molar formation and maintain the maxillary diastema as a tooth-free domain.

## Materials and methods

### Animals

Lhx7^+/LacZ^ embryos were generated by crossing heterozygous mice with C57BL/6 inbred animals. Lhx7 mutant embryos were generated by crossing homozygous males with heterozygous females. Double mutant embryos were collected from intercrosses of Lhx6^+/−^;Lhx7^+/LacZ^ mice maintained in a mixed background. In both cases, genotyping for the mutant loci was performed as described previously (Fragkouli et al., 2005; Liodis et al., 2007). For timed pregnancies, the day of vaginal plug was considered E0.5.

### Histology

For histological analysis, embryos of different developmental stages were either fixed in neutral buffered formalin, dehydrated and embedded in paraffin or fixed in 4% paraformaldehyde (PFA; in 1 × PBS), cryoprotected in 30% sucrose in 1 × PBS and embedded in 7.5% gelatin/15% sucrose in 1 × PBS. Paraffin sections (7 μm) and cryostat sections (10 μm) were stained with hematoxylin-eosin (H/E).

### Skeletal preparations

Staining of late embryonic mouse skeletons, has been performed according to the method of McLeod (1980). Briefly, E16.5 or E17.5 mouse embryos were skinned, eviscerated and fixed in 95% ethanol, dehydrated in acetone and stained using alizarin red S and alcian blue in acid ethanol. Following staining, the preparations were cleared in 1% potassium hydroxide (KOH) and finally stored in glycerol.

### BrdU incorporation

5-Bromodeoxyuridine (BrdU) stock solution (Sigma; 10 mg/ml) was made in 0.9% sodium chloride. This solution was injected intraperitoneally (10 μl/g of animal weight) into pregnant mice and embryos were harvested 2 h after the BrdU injection. Embryos were fixed for 2 h in 4% PFA at 4 °C and immunostained as described below.

### Analysis of cell death

TUNEL assay was performed using the Apop Tag *In Situ* Apoptosis Detection (Fluorescein) kit (Chemicon) by following the manufacturer's protocol. Apoptotic cells were also observed by immunohistochemistry for activated caspase-3, as described below.

### Detection of β-galactosidase (*lacZ*) activity

Whole embryos (E10.5–E12.5) or dissociated heads (E13.5–E15.5) were stained for β-galactosidase activity according to standard procedures. Embryos and heads were fixed in 1% PFA, 0.2% glutaraldehyde, 2 mM MgCl_2_, 5 mM EGTA, 0.02% NP-40 in PBS, for 30 and 60 min respectively. Fixed tissues were washed three times in 0.02% NP-40 in PBS and stained overnight (O/N) at room temperature (RT) using standard staining solution (5 mM K_3_Fe(CN)_6_, 5 mM K_4_Fe(CN)_6_^.^3H_2_O, 2 mM MgCl_2_, 0.01% NaDeoxycholate, 0.02% NP-40 and 1 mg/ml X-gal in PBS). The next morning the specimens were rinsed three times in 0.02% NP-40 in PBS and post-fixed in 4% PFA. Cryostat sections (10 μm) were used to observe *lacZ* expression at the cellular level. Some of the sections were counter-stained with eosin, while others were used to perform mRNA *in situ* hybridization, as described below.

### Immunohistochemistry

For immunohistochemistry, embryos were fixed in 4% PFA in PBS at 4 °C, O/N. Cryostat sections (10 μm) were permeabilized in 0.1% Triton X-100 in PBS (PBT) for 5 min, blocked in 1%BSA, 0.15% glycine in PBT for 1 h at RT and incubated with primary antibodies diluted in blocking solution at 4 °C, O/N. After washing three times with PBT, sections were incubated with secondary antibodies diluted in blocking solution at RT for 1 h. The following primary antibodies were used: rabbit polyclonal anti-phosphohistone-3 (Upstate Marker; 1/500), rat monoclonal anti-BrdU (Oxford Biotechnology/Serotec; 1/1000) and rabbit polyclonal anti-cleaved caspase-3 (Cell Signalling/New England Biolabs; 1/100). Secondary antibodies used are as follows: Alexa Fluor 488-conjugated goat anti-rabbit and Alexa Fluor 568-conjugated goat anti-rat (all from Invitrogen; all 1/500).

### RNA *in situ* hybridization

Non-radioactive whole mount or cryostat section (10 μm) mRNA *in situ* hybridization was performed as described previously (Riddle et al., 1993; Schaeren-Wiemers and Gerfin-Mose, 1993). Riboprobes used were specific for: *Activin-βA* (Feijen et al., 1994), *Barx1* (Tissier-Seta et al., 1995), *Bmp4* (Duprez et al., 1996), *EdaR* (Laurikkala et al., 2001), *Fgf8* (Crossley and Martin, 1995), *Gli1* (Marigo et al., 1996), *Gli2* (0.737 kb *Gli2* cDNA generated by PCR amplification, kindly provided by Dr. J. Briscoe), *Gli3* (Persson et al., 2002), *Gsc* (Blum et al., 1992), *Lhx6* (Grigoriou et al., 1998), *Lhx7* (Grigoriou et al., 1998), *Msx1* (MacKenzie et al., 1992), *p21*/*Waf1* (El-Deiry et al., 1993), *Pax9* (1.635 kb complete *Pax9* cDNA clone IMAGE:3707718), *Ptc1*/*2* (Pearse et al., 2001) and *Shh* (Echelard et al., 1993).

### Microscopy

Immunofluorescent sections were analysed with an epifluorescence microscope (Axiophot/Zeiss). All other samples were viewed with a Leica MZ 16 stereoscope (Leica). Brightfield photographs were taken with a QICAM 12-bit camera (Q Imaging) and analysed with Openlab.4 software.

### Quantification

The number of pH3 positive cells was evaluated in three E12.5 control and double mutant embryos. Briefly, two similar sized boxes have been defined in the “molar” mesenchyme of the maxilla and mandible, in control and Lhx6/7 KO embryos. We have counted the number of pH3 expressing cells found in the boxes in all serial histological sections corresponding to a single molar bud. To assess proliferation in the oral ectoderm we have counted pH3 positive cells in the epithelium that corresponds to the mesenchymal regions that were used for the previous measurements. Data are given as mean ± SE (standard error) and the statistical significance was based on the Student's test (*t*-test).

## Results

### Extensive co-expression of *Lhx6* and *Lhx7* in the first branchial arch, including the ectomesenchyme of the molar domain

**Fig. 1:**
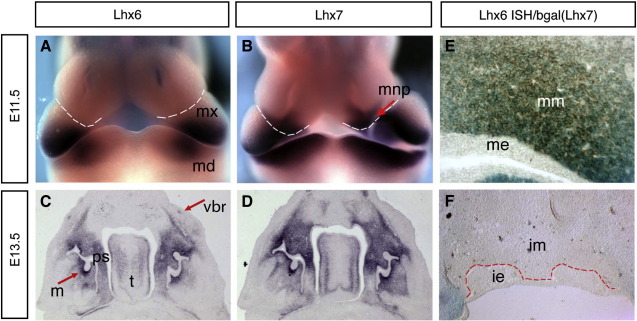
Lhx6 and Lhx7 are co-expressed in derivatives of the first branchial arch. Whole mount (A, B) or on sections of developing jaws (C, D) *in situ* hybridization for E11.5 (A, B) or E13.5 wild-type mouse embryos (C, D) using Lhx6- and Lhx7-specific riboprobes. Both genes are highly expressed in the oral mesenchyme of the maxillary (mx) and mandibular (md) processes. The developing nasal and maxillary processes in panels A and B are separated by a broken white line. The red arrow in panel B points to the *Lhx7* expression domain in the medial nasal process (mnp). In panels C and D teeth are at the bud stage of development. *Lhx6* and *Lhx7* are strongly expressed in the mesenchyme of molars (m), but also in the tongue (t) the palatal shelves (ps) and the follicles of vibrissae (vbr). Combination of *in situ* hybridization for *Lhx6* (brown) and β-gal histochemistry (blue) on sections of *Lhx7*^*+/LacZ*^ embryos at E11.5 (E) and E13.5 (F). Both genes are co-expressed at the single cell level in the molar mesenchyme (mm) underlying the molar epithelium (me) (E). In contrast no expression was detected in the incisor epithelium (ie) or mesenchyme (im) (F).

To address the possibility of redundant activity of *Lhx6* and *Lhx7* in the cranial mesenchyme, we first compared the expression profiles of the two genes in the head of wild-type embryos and identified domains in which they are co-expressed. For this analysis, we utilised Lhx6- and Lhx7-specific riboprobes and a transgenic mouse line (*Lhx7*^*LacZ*^) in which a β-galactosidase (β-gal) reporter is expressed under the control of the *Lhx7* locus and recapitulates its expression profile (Fragkouli et al., 2005). Consistent with previous reports, high levels of Lhx6 and Lhx7 mRNA were detected from E10.5 in the maxillary and mandibular processes of the first branchial arch and at E11.5, both genes were highly expressed in the ectomesenchyme adjacent to the oral cavity (Grigoriou et al., 1998, Tucker et al., 1999) (see also Fig. 1). Lhx6 mRNA was localized mainly in the proximal part of both the maxillary and mandibular processes while Lhx7 transcripts were detected along most of the proximal-distal axis of the mandibular and maxillary primordia, apart from the facial midline (Fig. 1, compare A with B). At this stage, expression of *Lhx7* was also detected in a few cells of the medial nasal process (Fig. 1B). At later stages (E13.5), the expression domains of *Lhx6* and *Lhx7* in the derivatives of the first branchial arch were highly overlapping: transcripts for both genes were present in the palatal shelves, the developing tongue and in vibrissae follicles (Fig. 1, compare C with D). Particularly high levels of expression for both genes were detected in ectomesenchymal cells condensing around the molar tooth buds (Figs. 1C, D). Moreover, by combining *in situ* hybridization (for *Lhx6*) and β-gal immunohistochemistry on cranial sections of E11.5 *Lhx7*^*+/LacZ*^ embryos, we observed that *Lhx6* and *Lhx7* were co-expressed in mesenchymal cells underlying the epithelial thickening of prospective molars (Fig. 1E). Interestingly, and in contrast to the molar domain, *Lhx6* and *Lhx7* were not expressed in mesenchymal cells associated with mandibular and maxillary incisors (Fig. 1F and data not shown). Together, these experiments demonstrate that the two genes are strongly co-expressed in cells of the molar mesenchyme.

### Maxillary and mandibular defects in Lhx6/7 double mutant embryos

**Fig. 2:**
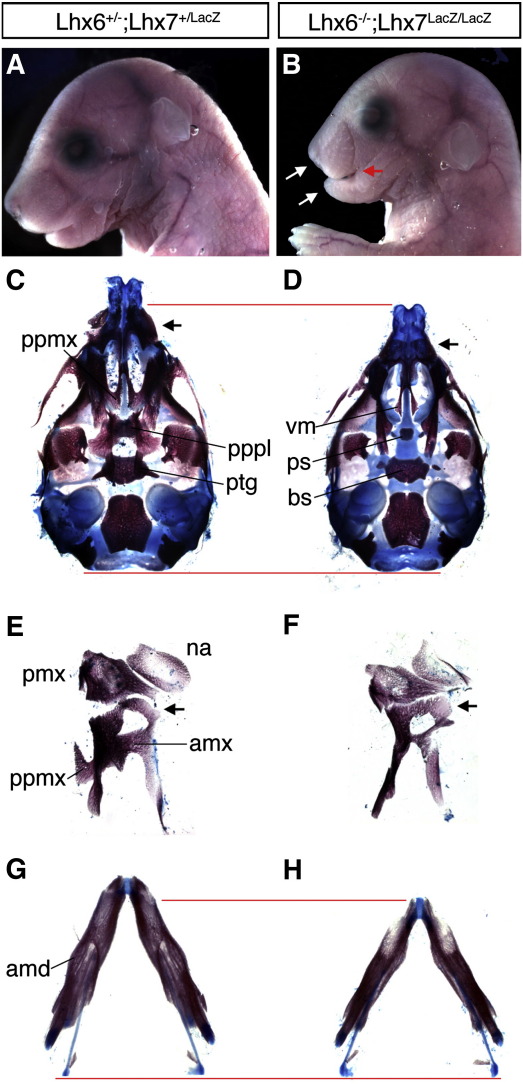
Craniofacial abnormalities in Lhx6/7-deficient mice. (A, B) Lateral profiles of the head of E16.5 control (A) and double mutant (B) embryos. The maxilla and the mandible of Lhx6/7 mutants are shortened along the proximal/distal axis (white arrows in B) and a gap between them is apparent (red arrow in B). (C, D) Ventral view of skeletal preparations of crania from E17.5 embryos after removal of the lower jaw. In control embryos (C) the palatal processes of the maxilla (ppmx) and the palatine (pppl) are clearly identified and in the process of fusion. In double mutant littermates (D), both palatal shelves are absent, allowing direct view of the vomer (vm) and the pre-sphenoid bones (ps). Lhx6/7-deficient mice are also characterized by absence of the pterygoid processes (ptg), which allows the view of the entire basisphenoid bone (bs). The general decrease in the size of the skull in double mutants is highlighted by red lines marking the anterior and posterior edges of the crania. (E, F) Ventral view of stained skeletal elements of the frontonasal (nasoethmoidal) region of control (E) and double mutant (F) E17.5 embryos. In double mutants both the palatal (ppmx) and alveolar (amx) processes of the maxilla are absent. The frontal (ascending) process of the maxilla is also abnormal (arrows in E and F) in morphology. (G, H) Rostral view of skeletal preparations of dissected mandibles from control (G) and double mutant (H) E17.5 embryos. The overall mandibular length is decreased in mutants (red lines mark the edges of the mandibles along the proximal/distal axis) while the alveolar bone (amd) surrounding the molar tooth cavity is absent. na, nasal bone; pmx, premaxilla.

To study the potential genetic interaction between *Lhx6* and *Lhx7* deletions and the combined role of these genes in the development of the first branchial arch, we inter-crossed double heterozygous mice (*Lhx6^+/−^*;*Lhx7*^*+/LacZ*^) to generate animals with all combinations of wild-type and mutant alleles. Phenotype analysis of *Lhx6^+/−^*;*Lhx7*^*+/LacZ*^ mice has shown that they develop normally, have no obvious morphological defects, are fertile and therefore have been used as controls in the studies described here. Consistent with the expression of *Lhx6* and *Lhx7* in the palate, mice bearing three mutant alleles (*Lhx6^−/−^*;*Lhx7*^*+/LacZ*^ or *Lhx6^+/−^*;*Lhx7*^*LacZ/LacZ*^) showed severe clefting of the secondary palate and died shortly after birth (data not shown). No other major craniofacial abnormalities were observed in these animals. In contrast, double homozygous mice (*Lhx6^−/−^*;*Lhx7*^*LacZ/LacZ*^; called hereafter double or Lhx6/7 mutants) were stillborn and, in addition to palatal clefting, had major craniofacial abnormalities. More specifically, double mutant embryos were recognizable from E13.5 by the significantly shorter mandibular and maxillary processes (micrognathia) relative to their littermate controls (data not shown). At later developmental stages (E16.5), double mutants showed a more dramatic reduction in the growth of the upper and lower jaws, which in these animals were separated by a diagnostic gap (Fig. 2, compare A with B).

To understand the anatomical basis of these defects, we compared whole mount skeletal preparations of heads from E17.5 double mutant and control embryos. Consistent with the complete clefting of the secondary palate, double mutants lacked both the palatal processes of the maxilla (ppmx) and the palatine (pppl) (Fig. 2, compare C with D), thus allowing direct visualization of the vomer (vm) and the presphenoid bone (ps; Fig. 2D). In addition to the lack of palatal processes, the alveolar process of the maxilla (amx), which surrounds the upper molar teeth, was absent (Fig. 2, compare E with F). The alveolar process of the mandible (amd) where the lower molar teeth are normally found was also absent from double mutant embryos (Fig. 2, compare G with H). Taken together, these findings show that progressive removal of functional *Lhx6* and *Lhx7* alleles results in increasingly abnormal development of neural crest-derived craniofacial derivatives.

### Combined deletion of *Lhx6* and *Lhx7* results in arrest of molar development at the initiation stage

**Fig. 3:**
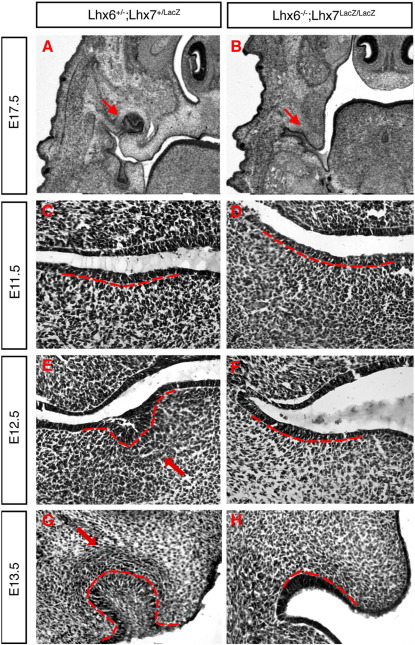
Absence of molars in double mutant mice. Transverse head sections processed for H/E staining from control (A, C, E, G) and mutant (B, D, F, H) embryos, at E17.5 (A, B), E11.5 (C, D), E12.5 (E, F) and E13.5 (G, H). In double mutant embryos, the molar epithelium and mesenchyme fail to undergo the normal morphogenetic changes associated with tooth formation, resulting in the absence of molars. The border between dental epithelium and mesenchyme is highlighted by a broken red line. Arrows point to the condensing molar mesenchyme in sections from control embryos (E and G). Such condensations are absent from equivalent sections from double mutant embryos (F and H).

The high levels of expression of *Lhx6* and *Lhx7* in the molar mesenchyme (Figs. 1C–E) suggested that the two genes have a role in odontogenesis. However, previous analysis of single mutant animals failed to show any significant defects in tooth formation (our unpublished observations). Moreover, histological sections of E17.5 mouse embryos carrying combinations of three mutant alleles of *Lhx6* and *Lhx7* showed that they develop both molars and incisors. In contrast, double mutant littermates reproducibly lacked molar teeth (Figs. 3A, B). To identify the stage at which molar development is affected, we have examined tooth development in histological sections of control (double heterozygous) and double mutant embryos from E11.5 to E17.5, a period encompassing many critical events of odontogenesis. As expected, localized thickenings of the oral epithelium, marking the sites of future molars, were observed in E11.5 control embryos (Fig 3C). In contrast, in double mutant littermates the oral epithelium of the molar domain showed no signs of thickening and maintained its uniform width (Fig. 3D). At E12.5, the thickening of the dental epithelium was clearly evident in control embryos with epithelial cells differentiating into dental placode and invading the underlying mesenchyme, which responds by characteristic condensation (Fig 3E). In contrast, in similar stage double mutant embryos, no evidence of thickening or invagination of the dental epithelium was observed and no corresponding condensation of the mesenchyme was evident (Fig. 3F). At E13.5, in contrast to control embryos in which molar development has reached the bud stage, no buds or any localized thickening of the dental epithelium was present in double mutant embryos (Fig. 3, compare G with H). Indeed, analysis of later embryonic stages showed that at no point in development did double mutant animals show clear signs of initiation of molar tooth formation (data not shown). Together, these studies demonstrate that in the absence of both *Lhx6* and *Lhx7* molar tooth development in mice is arrested at the initiation stage.

### Normal anterior–posterior patterning of the first branchial arch in Lhx6/7 mutant embryos

**Fig. 4:**
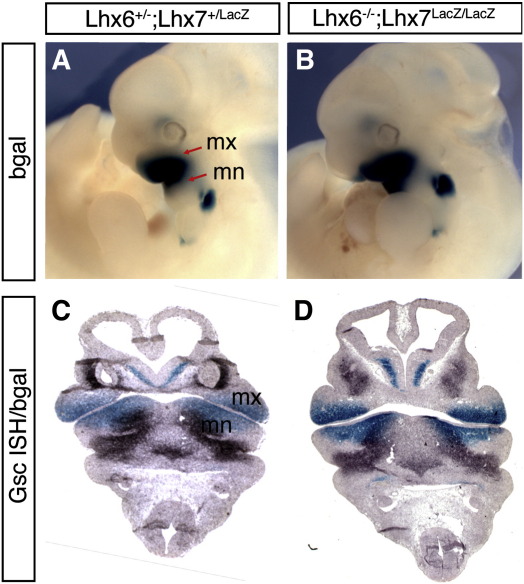
Normal oral/aboral patterning of the first branchial arch in Lhx6/7 mutants. (A, B) Whole mount β-gal staining for control (A) and double mutant (B) E11.5 embryos. The stronger signal in mutant relative to control embryos is due to homozygocity of the β-gal-expressing *Lhx7*^*LacZ*^ allele. The maxillary (mx) and mandibular (mn) processes are indicated (red arrows in A). (C, D) Combined *in situ* hybridization for *Gsc* and β-gal histochemistry (reflecting expression of *Lhx7*) in equivalent transverse sections from control (C) and Lhx6/7 deficient (D) E11.5 embryos. No differences are observed in the distribution of Gsc or β-gal-expressing cells between the two genotypes.

A potential explanation for the absence of molars in double mutant animals is that the oral–aboral patterning of the mandibular and maxillary primordia is altered and that in mutants the odontogenic (oral) mesenchyme has adopted an aboral (skeletogenic) character (Tucker et al., 1999). To test this hypothesis, we first compared the distribution of β-gal (a marker of Lhx7-expressing oral mesenchyme) in control and double mutant E11.5 embryos. B-gal staining was also compared to the expression pattern of Goosecoid (Gsc), a homeobox-transcription factor that is specifically expressed in the caudal domain of the mandible and is required for normal skeletogenesis in the head (Yamada et al., 1995; Rivera-Pérez et al., 1995). No difference in the distribution of βgal^+^ or Gsc-expressing cells was observed between control and double mutant embryos (Figs. 4A–D). These findings, together with the normal complement and position of maxillary and mandibular bones (Fig. 2) suggest that absence of molars from double mutant mice is unlikely to result from defects in the rostro-caudal patterning of the first branchial arch.

### Lhx6 or Lhx7 function is required for the specification of the odontogenic mesenchyme in the molar domain

**Supplement Fig. 1:**
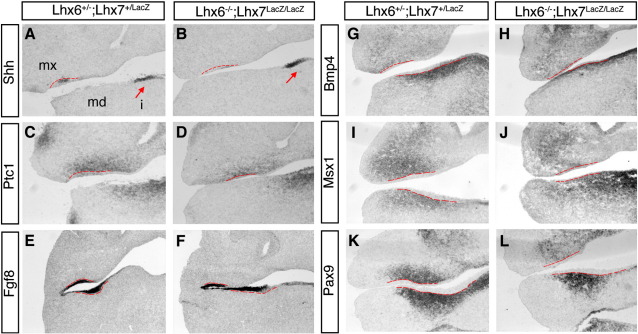
In double mutant mice expression of early mesenchymal and epithelial marker genes is maintained. *In situ* hybridization in transverse sections showing expression of markers genes in oral epithelium (*Shh*, *Fgf8*) and mesenchyme (*Ptc1*, *Bmp4*, *Msx1*, *Pax9*) of the molar domain of E11.5 control (A, C, E, G, I, K) and Lhx6/7-deficient (B, D, F, H, J, L) embryos. Dental epithelium is outlined in red. Red arrows in A and B indicate lower incisor tooth germs. mx, maxillary process; md, mandibular process.

The failure of initiation of molar development in Lhx6/7-deficient embryos could result from absence of inductive signals from the oral ectoderm or failure of the ectomesenchyme to respond to these signals. To explore these possibilities, we compared the expression of well-characterized epithelial and mesenchymal marker genes between mutant and control embryos up to the bud stage (E13.5). Fgf8 is an oral ectoderm-derived factor, which induces, in the underlying mesenchyme, expression of the transcription factors Pax9 and Barx, that in turn are critical for tooth development (Tucker et al., 1999; Trumpp et al., 1999; Mandler and Neubüser, 2001). Shh, a member of the vertebrate hedgehog family is also expressed in the oral ectoderm at the tooth initiation stage and its expression is restricted to the epithelial thickening of the early tooth germ (Dassule et al., 2000; Cobourne et al., 2001; Gritli-Linde et al., 2007). Effectors of Shh signalling, such as Ptc1 and Ptc2, and other known downstream targets, such as Gli1/2 and 3 proteins, are either expressed in the epithelium (Ptc2) or the underlying mesenchyme (Ptc1, Gli1/2/3) (Hardcastle et al., 1998). Finally, Bmp4, a member of the superfamily of TGFb signalling molecules, has a very dynamic expression pattern during tooth development. Before the dental lamina stage it is expressed in the oral epithelium but shortly afterwards, its expression shifts to the mesenchyme where it is responsible for the induction of the homeodomain transcription factor Msx1 (Aberg et al., 1997; Vainio et al., 1993). *In situ* hybridization on transverse sections from E11.5 control and double mutant embryos revealed that all of the above marker genes are expressed in both genotypes (Supplement Figs. 1A–L). Therefore, the combined deletion of *Lhx6* and *Lhx7* does not prevent the oral ectoderm to produce the signals associated with initiation of odontogenesis. In addition, our findings suggest that the ectomesenchyme of the molar domain is capable of responding, at least partly, to the earliest odontogenic signals.

**Supplement Fig. 2:**
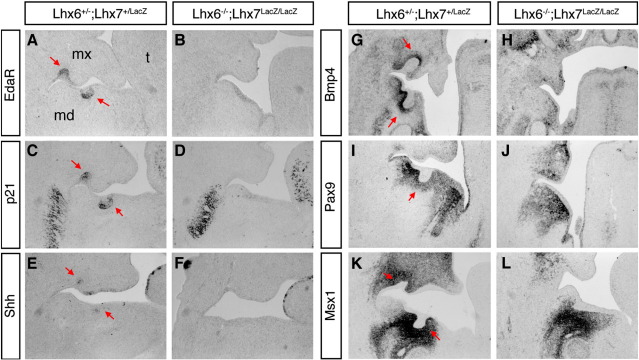
Molar development in Lhx6/7 mutant embryos is permanently arrested at the dental lamina stage. *In situ* hybridization in transverse sections for marker genes of dental placodes (*Shh*, *EdaR*, *p21*) and mesenchyme (*Bmp4*, *Msx1* and *Pax9*) in the molar domain of E13.5 control (A, C, E, G, I, K) and double mutant (B, D, F, H, J, L) embryos. At E13.5, molars are at the bud stage in control mouse embryos (red arrows in A, C, E, G, I, K), in contrast, no tooth buds are present in the molar domain of double mutant mice (B, D, F, H, J, L). In addition, EdaR (B), p21 (D) and Shh (F) transcripts are absent in the dental epithelium, while Bmp4 (H) and Msx1 (L) transcripts are barely detectable in the mesenchyme in the “molar” domain of Lhx6/7 deficient mouse embryos. Pax9 transcripts are detected both in control and double mutant embryos (I and J respectively). Note that mRNA transcripts are detected for all markers in areas outside the molar domain irrespective of genotype. mx, maxillary process; md, mandibular process; t, tongue.

**Fig. 5:**
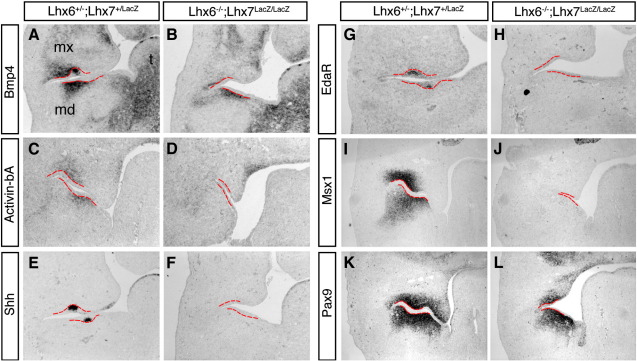
Absence of dental placodes and failure of specification of odontogenic mesenchyme in the molar domain of Lhx6/7 double mutant embryos. *In situ* hybridization in transverse cranial sections through the molar domain of E12.5 control (A, C, E, G, I, K) and double mutant (B, D, F, H, J, L) embryos. Note the absence of Shh (F) and EdaR (H) transcripts from the molar epithelium in double mutant embryos. In addition, expression of Bmp4 (B), Activin-βA (D) and Msx1 (J) is severely reduced in the mesenchyme of Lhx6/7 mutants relative to controls. The border between dental epithelium and mesenchyme is shown by a red broken line. mx, maxillary process; md, mandibular process; t, tongue.

Induction of several transcription factors and signalling molecules in the mesenchyme of odontogenic sites at E11.5 marks a shift of inductive potential from the oral ectoderm to the underlying mesenchyme (Mina and Kollar, 1987) and is followed by the expression of reciprocal inductive signals by the adjacent oral epithelium. Two such mesenchymal signals, BMP4 and Activin βA (members of the TGFβ family of signalling molecules) induce overlying epithelial cells to form transient signalling centres, the dental placodes (Jernvall and Thesleff, 2000). Among the genes that are expressed at the dental placodes are *Shh*, *p21* and *EdaR*, which encode the receptor of ectodysplasin (EDA), a tumor necrosis factor family member that is also expressed in the dental placode (Jernvall et al., 1998). To investigate whether the molar ectomesenchyme of double mutant embryos has been properly specified as odontogenic and thus is competent to produce signals that further advance tooth formation, we compared the expression of several marker genes in the molar region of E12.5 control and mutant embryos by *in situ* hybridization. In control embryos, Bmp4 and Activin βA transcripts are found in mesenchymal cells underlying the forming epithelial buds (Figs. 5A, C). In contrast, expression of both genes in double mutants was either severely diminished or completely absent (Figs. 5B, D). Consistent with the reduced expression of these signalling molecules, E12.5 double mutant embryos showed a dramatic down-regulation of dental placode markers, such as Shh, EdaR and p21, relative to control littermates (Figs. 5E–H and data not shown). Interestingly, expression of *Msx1* in double mutants was also severely reduced (Figs. 5I, J), but Pax9 transcripts were detected in the mesenchyme of both control and double mutant embryos, although its expression domain in mutants was significantly reduced (Figs. 5K, L). Similar changes of marker gene expression were also observed in the molar domain of double mutant embryos analysed at E13.5 (Supplementary Figs. 2, A–L). Finally, no differences were observed in the expression pattern of *Fgf8* and *Barx1* in sections from double mutant relative to control embryos (data not shown). Taken together, these findings suggest that deletion of *Lhx6* and *Lhx7* results in failure of normal differentiation of molar mesenchyme leading to the arrest of molar development at the dental lamina stage.

### Combined deletion of *Lhx6* and *Lhx7* results in increased cell death and reduced proliferation in first branchial arch derivatives

**Fig. 6:**
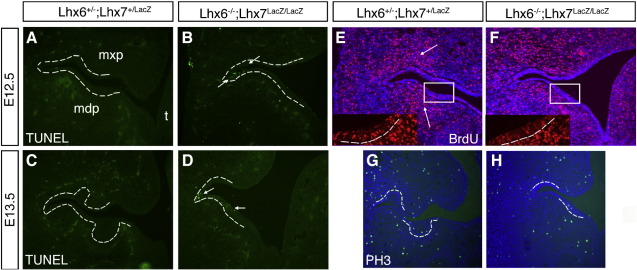
The survival and proliferation of epithelial and mesenchymal cells in the molar domain of Lhx6,7 double mutant embryos is compromised. (A–D) TUNEL assay in equivalent transverse sections from E12.5 (A, B) and E13.5 (C, D) control (A, C) and double mutant (B, D) embryos. There is no obvious apoptotic activity within the oral epithelium or mesenchyme in control sections at both developmental stages (A, C). However, in Lhx6/7-deficient mouse embryos apoptotic cells were detected primarily in the oral epithelium (white arrows) (B, D). (E, F) Sections from E12.5 control (E) and double mutant (F) embryos labelled with BrdU shortly before harvesting. Insets in panels E and F represent magnifications of the indicated regions. Relative to control sections, fewer BrdU^+^ cells were observed both in the dental epithelium and the mesenchyme of the molar domain of double mutant embryos. (G, H) pH3 immunofluorescence on sections from control (G) and double mutant (H) embryos. The number of pH3 positive cells both in the oral epithelium and mesenchyme of the molar domain is reduced in Lhx6/7 deficient (H) relative to control (G) animals. Oral ectoderm is outlined by a white broken line. (mx, maxillary process; md, mandibular process; (t) tongue.

The failure of dental placode/dental lamina formation and the lack of condensation of the underlying ectomesenchyme could result from increased cell death or impaired proliferation. To examine these possibilities, we first analysed apoptotic cell death in the maxillary and mandibular arches of control and double mutant embryos at E12.5 and E13.5 using TUNEL staining. Relative to double heterozygous controls, Lhx6/7-deficient embryos showed a dramatically increased number of apoptotic cells in the oral epithelium of both the maxilla and the mandible (Figs. 6A–D). Interestingly, the increased apoptotic activity was primarily observed in the dental lamina, although apoptotic cells could also be seen in other regions of the oral ectoderm of double mutants (arrows in Figs. 6B, D).

We also assessed cell proliferation in the maxillary and mandibular primordia of the first branchial arch, using immunostaining for phosphohistone-3 (pH3) and in vivo BrdU pulse labelling. This analysis showed that the number of pH3 positive cells detected in the maxillary and mandibular epithelium on transverse sections from double mutant E12.5 embryos (3.33 ± 0.57 and 4.0 ± 1.0, respectively) was dramatically reduced relative to control littermates (11.33 ± 0.57 and 12.0 ± 2.0, respectively; *P* < 0.005). A significant reduction of mitotic cells was also observed in the maxillary mesenchyme of mutant (12.66 ± 1.15) relative to control embryos (22.0 ± 2.0; *P* < 0.05), whereas the proliferative defect in the mandibular mesenchyme was less prominent (15.0 ± 2.0 in mutants vs. 19.66 ± 2.52 in controls; *P* = 0.07) (Figs. 6G, H). Similarly, BrdU labelling was reduced in the molar mesenchyme as well as the dental lamina of double mutants relative to littermate controls (Figs. 6E, F). These results show that both survival and proliferation of molar mesenchyme and dental epithelium are compromised in embryos lacking Lhx6 and Lhx7.

### Normal mandibular but supernumerary maxillary incisors in mice lacking *Lhx6* and *Lhx7* functions

**Fig. 7:**
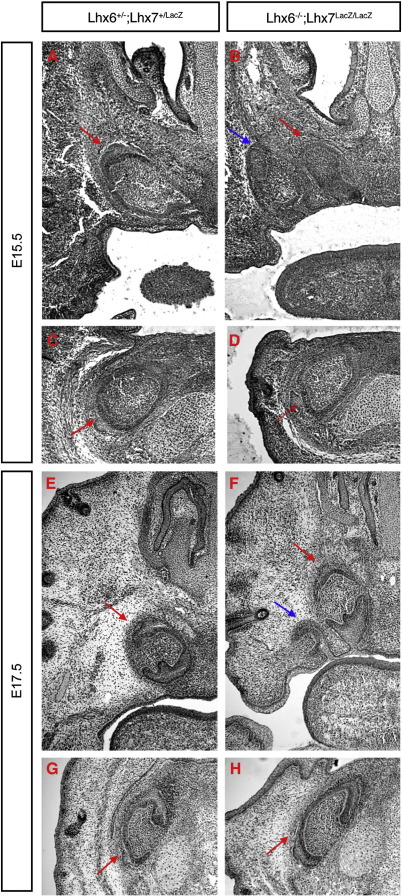
Normal development of incisors but supernumerary teeth in the maxilla of Lhx6/7 mutant embryos. Histological sections from E15.5 (A–D) and E17.5 (E–H) control (A, C, E, G) and double mutant (B, D, F, H) mice. Maxillary (A, B, E, F) and mandibular (C, D, G, H) incisors (red arrows) develop normally in both control (A, C, E, G) and Lhx6/7-deficient (B, D, F, H) mouse embryos. Note that supernumerary incisor-like teeth develop laterally to the original maxillary incisors in double mutant embryos (blue arrows in B and F).

In contrast to the proximal (molar) domain, *Lhx6* and *Lhx7* are not expressed in the most distal part of the maxillary and mandibular primordia, where upper and lower incisors form, respectively. Given the absence of molars and the abnormal development of the maxillary process, which has been suggested to affect distal odontogenesis in rodents (Peterková et al., 1993, Kriangkrai et al., 2006), we wished to examine the effect of combined deletion of *Lhx6* and *Lhx7* on the development of incisors. Histological sections showed that at E15.5 incisor development had reached the cap stage in both control and double mutant embryos and no obvious morphological differences were observed between the two genotypes (Figs. 7A, B). Surprisingly, but reproducibly, Lhx6/7 mutant embryos possessed supernumerary teeth in the maxilla (blue arrow in Fig. 7B).These extra teeth were present laterally, were somewhat smaller and rotated through 90° with respect to the normal incisors. Analysis at later developmental stages (E17.5) confirmed the results obtained at E15.5, namely showing normal development of maxillary incisors and supernumerary pair of teeth (Figs. 7E, F; blue arrow in panel F points to the extra tooth). In no case did we observe additional teeth in the mandibular process (Figs. 7C, D and G, H). These data show that, deletion of *Lhx6* and *Lhx7* does not effect incisor development but results in the presence of supernumerary teeth specifically in the incisor domain of the maxilla.

**Fig. 8:**
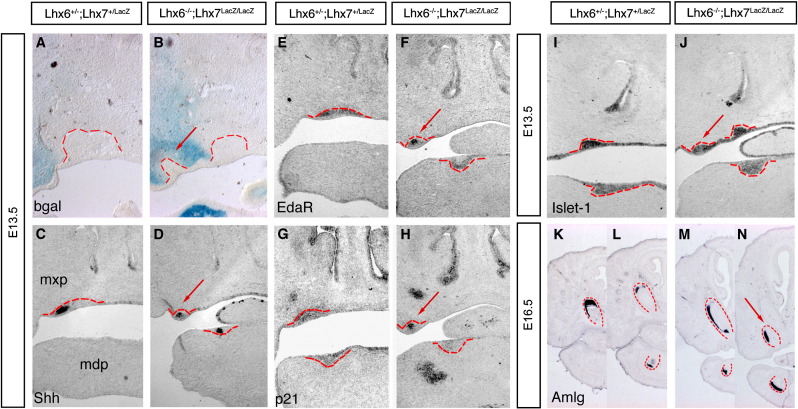
The supernumerary upper incisors of double mutant embryos arise from distinct dental placodes embedded in the diastema region of the maxilla. β-gal staining (A, B) and *in situ* hybridization for marker gene analysis (C–J) in transverse sections from control (A, C, E, G, I) and Lhx6/7 deficient (B, D, F, H, J) E13.5 embryos. (K–N) *In situ* hybridization for Amelogenin (Amlg) in frontal sections from E16.5 control (K, L) and double mutant (M, N) embryos. L and N represent more posterior sections relative to those shown in K and M. Dental epithelium is separated from the underlying mesenchyme by a red broken line. Red arrows in B, D, F, H, J and N indicate the supernumerary incisors. Panels D, F and H represent sections that contain only the lateral supernumerary dental placode. Mx, maxillary process; md, mandibular process.

To explore the mechanisms underlying the formation of supernumerary teeth in Lhx6/7 double mutant embryos, we carried out histological analysis of maxillae at early stages of odontogenesis. In E13.5 controls, a single dental placode was formed in the most distal part of each dental quadrant of the maxillary process, and begun to invaginate to the underlying, condensed mesenchyme (Fig. 8A). In contrast, double mutant littermates reproducibly showed two epithelial thickenings invading the mesenchyme (Fig. 8B). The lateral (supernumerary) invaginations (red arrow in Fig. 8B) were smaller than the medial ones and were surrounded by βgal^+^ mesenchymal cells, suggesting that, contrary to the normal incisors, they form in an Lhx7-expressing domain of the distal ectomesenchyme which normally does not form teeth (diastema region). To obtain further evidence that the supernumerary epithelial thickenings observed in the maxillary primordium of double mutants were odontogenic placodes, we examined the expression of Shh, p21 and EdaR (see above). As expected, these markers were expressed strongly in the normal incisor placodes of both control and double mutant embryos (Figs. 8C–H). Importantly, these markers were also expressed by epithelial cells of the more lateral (supernumerary) invaginations, indicating that they represent bona fide odontogenic placodes (Figs. 8D, F, H; red arrows point to supernumerary teeth). We conclude that the formation of the extra teeth observed in the maxilla of double mutant embryos, recapitulates the morphological and molecular stages that are observed during normal odontogenesis.

Examination of histological sections from the maxilla of E17.5 double mutant embryos suggests that the supernumerary teeth had morphological characteristics of incisors. To explore further the identity of these supernumerary dental placodes, we analysed the expression of Islet1, a LIM homeodomain transcription factor that is expressed in the distal oral epithelium of the first branchial arch, including that of the incisor placodes, but is not detected in the epithelial cells of the molar domain (Mitsiadis et al. 2003). As shown in Fig. 8 (I, J), Isl1 transcripts were present in the distal domain of the maxilla in both control and double mutant embryos, including the supernumerary epithelial thickenings of Lhx6/7-deficient animals. To provide further support for the incisor identity of supernumerary teeth, we analysed the expression of Amelogenin (Amlg) in sections of E16.5 control and double mutant embryos. Amelogenins are matrix proteins synthesized and secreted by pre-ameloblasts during differentiation stage (late bell stage) and ameloblasts during secretory stage, and contribute to the formation of the dental enamel (Karg et al., 1997). In contrast to molars, incisors display a characteristic asymmetric expression pattern of Amelogenin, namely restriction to the labial side of the cervical loop. We observed that, similar to the normal incisors of control (Figs. 8K, L) and double mutant embryos (Fig. 8M), Amelogenin transcripts were observed specifically in the labial side of the supernumerary teeth of Lhx6/7-deficient embryos, as well (Fig. 8N). Taken together, these findings suggest that the supernumerary teeth observed in double mutant animals are incisors, which arise from distinct dental placodes that are located laterally to the site of normal incisor placodes and are embedded within the Lhx7-expressing diastemal mesenchyme.

## Discussion

In the present report we have analysed the role of the LIM homeodomain transcription factors Lhx6 and Lhx7 in murine dentition. Despite the robust expression of *Lhx6* and *Lhx7* in neural crest-derivatives of the first branchial arch (Grigoriou et al., 1998), mice homozygous for single gene deletions show no defects in the formation of teeth or other cranial skeletal structures, apart from cleft palate that has been observed in a percentage (approx. 70%) of Lhx7-deficient animals (Zhao et al., 1999; our observations). Here, we demonstrate that both genes are co-expressed widely in the first branchial arch and its derivatives, including the palatal shelves and the molar domain of the maxillary and mandibular processes. Consistent with this expression pattern all compound mutant animals with three deletion alleles (i.e. Lhx6^−/−^;Lhx7^+/LacZ^ or Lhx6^+/−^;Lhx7^LacZ/LacZ^) show severe clefting of the palate, indicating a dosage-dependent and partially redundant activity of *Lhx6* and *Lhx7* genes. Similar redundant functions of Lhx6 and Lhx7 in molar tooth formation are also evident by the failure of molar formation in Lhx6/7-deficient animals. However, this phenotype was manifested only in mice lacking all four wild-type alleles, demonstrating that a single functional copy of either *Lhx6* or *Lhx7* is sufficient to support normal development of molars. Although we cannot exclude the possibility that single mutants or animals with three mutant alleles have subtle tooth abnormalities, our studies suggest that the activity of the two genes in the molar mesenchyme is interchangeable and that relatively low levels of either factor are sufficient to support molar odontogenesis. Despite the absolute requirement of *Lhx6* or *Lhx7* activity for molar development, these genes are not required for incisor formation, an observation consistent with their minimal expression in the incisor domain of the ectomesenchyme. In fact, deletion of both genes reproducibly leads to extra incisor tooth formation specifically in the maxilla.

Branchial arches develop along a characteristic oral–aboral (rostral-caudal) axis, which is evident by the anatomical landmarks of the derivative head structures. Therefore, the absence of molars in Lhx6/Lhx7-deficient animals could result from a patterning defect, which disrupts the oral–aboral axis of the first branchial arch resulting in failure of formation of odontogenic mesenchyme. Signals from the oral ectoderm, such as Fgf8, has been reported to be primarily responsible for co-ordinating this polarity through the early induction of its target genes *Lhx6* and *Lhx7* in the oral domain of the first branchial arch, prior to the initiation of odontogenesis, which in turn restrict the expression of Gsc in the caudal (skeletogenic) domain (Tucker et al., 1999). Based on these findings, we previously proposed that Lhx6 and Lhx7 may be part of the molecular cascade that sets-up the oral–aboral axis of the maxillary and mandibular processes of the first branchial arch thus leading to the asymmetric formation of odontogenic ectomesenchyme and teeth (Grigoriou et al., 1998; Tucker et al., 1999). Here we have analysed animals with a combined deletion of *Lhx6* and *Lhx7* and found no evidence of changes in the patterning of the first branchial arch. Thus, we observed a normal distribution of characteristic markers of the anterior (such as β-gal^+^) or posterior ectomesenchyme (Gsc) in double mutant embryos at E11.5, while analysis of histological sections or skeletal preparations of Lhx6/Lhx7 double mutants produced no evidence of ectopic bone formation in tooth regions. Finally, the correct rostro-caudal patterning of the first branchial arch in the Lhx6/7 double mutants is supported by the development of incisors in both the maxillary and mandibular processes. We conclude that upon deletion of *Lhx6* and *Lhx7*, oral ectomesenchymal cells cannot be re-specified as chondrogenic, suggesting that other factors are primarily responsible for establishing and maintaining the anterior–posterior axis of the first branchial arch.

Despite the failure of molar formation, combined deletion of *Lhx6* and *Lhx7* does not prevent initiation of molar odontogenesis. This is supported by the observation that critical inductive signals, such as Fgf8, Shh and Bmp4 are produced by the oral epithelium of double mutant embryos. Furthermore, the underlying ectomesenchyme in Lhx6^−/−^;Lhx7^LacZ/LacZ^ embryos is capable of responding to the initiating epithelial signals of the odontogenic domains. Thus, Ptc1 and Gli1/2/3 are induced in the mesenchyme, indicating an intact Shh pathway, while Fgf8 is capable of inducing expression of the *Lhx7*^*LacZ*^ allele (as evidenced by the presence of βgal^+^ expressing cells) and the *Barx1*, *Pax9* and *Dlx1* genes. Finally, *Msx1*, a target of both Bmp4 and Fgf8 (Vainio et al., 1993; Bei and Maas, 1998), is also induced in the odontogenic mesenchyme. These mesenchymal transcription factors control the expression of reciprocal signals to the epithelium, which in turn responds by budding to the underlying ectomesenchyme and forming transient signalling centres, the dental placodes. Two such mesenchyme-derived signals, Bmp4 and Activin bA, have been identified as major mediators of the inducing activity of the mesenchyme at E12.0 (Mina and Kollar, 1987). Interestingly, expression of both *Bmp4* and *Activin bA* are dramatically reduced in the mesenchyme of E12.5 Lhx6^−/−^;Lhx7^LacZ/LacZ^ embryos resulting in failure of induction of target genes, such as *Shh*, *p21* and *Edar* (Jernvall and Thesleff, 2000; Zhang et al., 2000), in the overlying epithelial cells. The absence of critical signalling molecules within the oral epithelium could provide an explanation for the failure of dental lamina to progress to the bud stage. In particular, Shh has been proposed to promote cell survival and proliferation of the oral ectoderm, which are obvious prerequisites of growth and morphogenesis of dental epithelium. Consistent with these studies, we have observed increased cell death and reduced cell proliferation in the dental lamina of Lhx6/Lhx7 double mutant animals. Although it is currently unclear whether the activity of Lhx6 and Lhx7 within the mesenchyme is exclusively mediated by epithelial Shh, our finding demonstrates that either of these factors is required for the proximal maxillary and mandibular mesenchyme to acquire its odontogenic capacity and signal to the epithelium to induce formation of molar placodes.

Several transcription factors have been shown to have critical roles in tooth development. Thus, double knockout mice for Msx1 and Msx2, Gli2 and Gli3, and Dlx1 and Dlx2 are also characterized by arrest of tooth development at early stages of odontogenesis (Jernvall and Thesleff, 2000). However, these genetic combinations result in a phenotype that is distinct from that of the Lhx6/7 double mutant mice. More specifically, Msx1/Msx2-defcient embryos do not generate recognizable tooth buds (Bei and Maas, 1998; Satokata et al., 2000) while Gli2/Gli3 mutant mice show absence of molars and severe retardation of incisor development (Hardcastle et al., 1998). Similar to Lhx6/7 double mutants, deletion of Dlx1 and Dlx2 results in specific deficits of molar formation, but this phenotype is restricted to the maxilla, presumably due to the redundant activity of *Dlx5*/*6* genes which are expressed in the ectomesenchyme of the mandibular primordium (Thomas et al., 1997). Finally, in the absence of Dlx1 and Dlx2, maxillary molar odontogenic mesenchyme adopts a partial chondrogenic potential, a transformation that has not been observed in Lhx6^−/−^;Lhx7^LacZ/LacZ^ mutant mice. Taken together with our present findings, these studies suggest that the Lhx6 and Lhx7 LIM homeodomain proteins control unique aspects of molar and incisor tooth development.

It is likely that complex co-ordinated activities of multiple transcription factors, some of which are likely to be direct or indirect targets of Lhx6 and Lhx7, are necessary for developing teeth to progress from the dental lamina to the bud stage. As an illustrating example of such complex interactions, it has been recently demonstrated *in vitro*, that Pax9 directly regulates the expression of *Msx1* but also interacts with its product to enhance its ability to trans-activate the *Msx1* and *Bmp4* genes during tooth development (Ogawa et al., 2006). Our present findings demonstrate that the expression of *Msx1*, but not *Pax9*, was down-regulated in Lhx6/7 double mutant mice, at E12.5. As induction of *Msx1* (up to E11.5) was not affected in Lhx6/7-deficient mice, it is unlikely that Lhx6 or Lhx7 are involved in the transcriptional activation of this locus; instead our findings suggest that these LIM homeodomain proteins may have a key role in maintaining the expression of *Msx1* in molar ectomesenchyme. Overall, our current genetic studies suggest that the Lhx6 and Lhx7 LIM homeodomain proteins are key components of a transcriptional network that controls the acquisition of odontogenic potential by molar mesenchyme.

One of the most salient features of the dental phenotype of Lhx6/Lhx7 double mutant mice is the appearance of an extra pair of teeth, lateral to the original incisors. Based on morphological and molecular criteria, the additional teeth appear to be incisors that are generated from individual dental placodes, which contrary to the original incisors, form in an Lhx7 expressing domain of the ectomesenchyme. Rodents normally have fewer teeth than most mammals, with only one incisor separated by a toothless diastema region from three molars, in each dental quadrant. The genetic and molecular mechanisms controlling this dentition pattern are currently unknown. In general, the number of tooth primordia generated during mouse embryogenesis in the maxilla is higher compared to the final number of teeth (Peterková et al., 2002). In the incisor domain, primary dental placodes normally fuse to form a single composite upper incisor primordium (Peterková et al., 1993; Kriangkrai et al., 2006). In contrast, in the upper diastema, dental primordia are eliminated by apoptosis (Peterková et al., 2002). These observations suggest that the supernumerary incisors in the maxilla of Lhx6/Lhx7 mutant embryos result either from the failure of fusion of the primordial epithelial thickenings or from persistence of diastemal dental primordia.

Both mice and rats with mutations of the *Pax6* locus have been reported to have excessive number of maxillary incisors (Kaufman et al., 1995, Quinn et al., 1997). More specifically, approximately 90% of homozygous *Small eye* (*Pax6Sey*) mice possess one or two supernumerary teeth adjacent to the original incisors (Kaufman et al., 1995). Although no other dental defects have been described in these mutants, they are also characterized by cranial skeletal abnormalities, such as absence of nasal derivatives, raising the possibility that the dental patterning defects are secondary to the primary skeletal abnormalities. In support of this view, a recent study of rats homozygous for the spontaneous *Pax6* mutation *rSey2*, demonstrated a significant incidence of extra upper incisors (25%) and suggested that they form as a result of a persistent cleft between the medial nasal and maxillary processes which inhibits their fusion and maintains two independent dental placodes (Kriangkrai et al. 2006). Although Lhx6/Lhx7 mutants do not lack nasal structures and do not develop permanent clefts in the frontonasal region, they are characterised by reduced maxillary growth which results in morphological abnormalities of the frontofacial region, where upper incisors form. It is therefore possible that lack of co-ordinated growth of the different components of the upper jaw in Lhx6/Lhx7-deficient mice, results in the maintenance of two additional dental placodes in each quadrant, which subsequently develop into two distinct incisors. In an alternative model, supernumerary incisors could originate from diastemal dental placodes that fail to be eliminated in double mutant mice. The potential mechanisms underlying the persistence of dental placodes are currently unknown, but our analysis clearly shows that the additional maxillary teeth develop in a domain that normally expresses high levels of *Lhx7* but generally undetectable levels of Lhx6. Although this observation suggests that the activity of Lhx7 alone could prevent the progression of odontogenesis in the diastema region of the maxilla, this view is not supported by the analysis of single Lhx7 mutants that show lack of extra incisors. Moreover, analysis of Lhx6 expression in Lhx7 mutants failed to detect increased levels of Lhx6 in the maxillary diastema (data not shown). It is possible that the inhibitory role of LIM homeodomain factors on the odontogenic activity of the maxillary diastema involves both Lhx6 and Lhx7, and that these molecules have both cell autonomous and non-cell autonomous effect. Such a transformation in the odontogenic potential may as well be happening in the mandibular diastema of double mutants, but could not be manifested by the presence of extra incisors since no transient dental primordia have been described in the diastema of mouse mandible (Peterková et al., 2002). Although the molecular details by which Lhx6 and Lhx7 inhibit odontogenesis in the diastema region while promoting tooth formation in the nearby molar domain remain unclear, our experiments uncover a specific requirement of these transcription factors in molar formation and reveal a role in co-ordinating molar odontogenesis with the patterning of incisor dentition.

## References

[bib1] Aberg T., Wozney J., Thesleff I. (1997). Expression patterns of bone morphogenetic proteins (Bmps) in the developing mouse tooth suggest roles in morphogenesis and cell differentiation. Dev. Dyn..

[bib2] Arber S., Caroni P. (1996). Specificity of single LIM motifs in targeting and LIM/LIM interactions in situ. Genes Dev..

[bib3] Bach I. (2000). The LIM domain: regulation by association. Mech. Dev..

[bib4] Bei M., Maas R. (1998). FGFs and BMP4 induce both Msx1-independent and Msx1-dependent signaling pathways in early tooth development. Development.

[bib5] Blum M., Gaunt S.J., Cho K.W., Steinbeisser H., Blumberg B., Bittner D., De Robertis E.M. (1992). Gastrulation in the mouse: the role of the homeobox gene goosecoid. Cell.

[bib6] Chai Y., Maxson R.E. (2006). Recent advances in craniofacial morphogenesis. Dev. Dyn..

[bib7] Cobourne M.T., Sharpe P.T. (2003). Tooth and jaw: molecular mechanisms of patterning in the first branchial arch. Arch. Oral Biol..

[bib8] Cobourne M.T., Hardcastle Z., Sharpe P.T. (2001). Sonic hedgehog regulates epithelial proliferation and cell survival in the developing tooth germ. J. Dent. Res..

[bib9] Courtney J.-M., Blackburn J., Sharpe P.T. (2005). The ectodysplasin and NFkappaB signalling pathways in odontogenesis. Arch. Oral Biol..

[bib10] Crossley P.H., Martin G.R. (1995). The mouse *Fgf8* gene encodes a family of polypeptides and is expressed in regions that direct outgrowth and patterning in the developing embryo. Development.

[bib11] Curtiss J., Heilig J.S. (1995). Establishment of *Drosophila* imaginal precursor cells is controlled by the *Arrowhead* gene. Development.

[bib12] Dassule H.R., Lewis P., Bei M., Maas R., McMahon A.P. (2000). Sonic hedgehog regulates growth and morphogenesis of the tooth. Development.

[bib13] Duprez D., Bell E.J., Richardson M.K., Archer C.W., Wolpert L., Brickell P.M., Francis-West P.H. (1996). Overexpression of BMP-2 and BMP-4 alters the size and shape of developing skeletal elements in the chick limb. Mech. Dev..

[bib14] Echelard Y., Epstein D.J., St-Jacques B., Shen L., Mohler J., McMahon J.A., McMahon A.P. (1993). Sonic hedgehog, a member of a family of putative signaling molecules, is implicated in the regulation of CNS polarity. Cell.

[bib15] El-Deiry W.S., Tokino T., Velculescu V.E., Levy D.B., Parsons R., Trent J.M., Lin D., Mercer W.E., Kinzler K.W., Vogelstein B. (1993). WAF1, a potential mediator of p53 tumor suppression. Cell.

[bib16] Feijen A., Goumans M.J., van den Eijnden-van Raaij A.J. (1994). Expression of activin subunits, activin receptors and follistatin in postimplantation mouse embryos suggest specific developmental functions for different activins. Development.

[bib18] Fragkouli A., Hearn C., Errington M., Cooke S., Grigoriou M., Bliss T., Stylianopoulou F., Pachnis V. (2005). Loss of forebrain cholinergic neurons and impairment in spatial learning and memory in LHX7-deficient mice. Eur. J. Neurosci..

[bib19] Grigoriou M., Tucker A.S., Sharpe P.T., Pachnis V. (1998). Expression and regulation of Lhx6 and Lhx7, a novel subfamily of LIM homeodomain encoding genes, suggests a role in mammalian head development. Development.

[bib20] Gritli-Linde A., Hallberg K., Harfe B.D., Reyahi A., Kannius-Janson M., Nilsson J., Cobourne M.T., Sharpe P.T., McMahon A.P., Linde A. (2007). Abnormal hair development and apparent follicular transformation to mammary gland in the absence of hedgehog signaling. Dev. Cell.

[bib21] Hardcastle Z., Mo R., Hui C.C., Sharpe P.T. (1998). The Shh signalling pathway in tooth development: defects in Gli2 and Gli3 mutants. Development.

[bib22] Hobert O., Westphal H. (2000). Functions of LIM-homeobox genes. Trends Genet..

[bib24] Jernvall J., Thesleff I. (2000). Reiterative signaling and patterning during mammalian tooth morphogenesis. Mech. Dev..

[bib25] Jernvall J., Aberg T., Kettunen P., Keränen S., Thesleff I. (1998). The life history of an embryonic signaling center: BMP-4 induces p21 and is associated with apoptosis in the mouse tooth enamel knot. Development.

[bib26] Karg H.A., Burger E.H., Lyaruu D.M., Wöltgens J.H., Bronckers A.L. (1997). Gene expression and immunolocalisation of amelogenins in developing embryonic and neonatal hamster teeth. Cell Tissue Res..

[bib27] Kaufman M.H., Chang H.H., Shaw J.P. (1995). Craniofacial abnormalities in homozygous Small eye (Sey/Sey) embryos and newborn mice. J. Anat..

[bib28] Kitanaka J., Takemura M., Matsumoto K., Mori T., Wanaka A. (1998). Structure and chromosomal localization of a murine LIM/homeobox gene, *Lhx8*. Genomics.

[bib29] Klein O., Minowada G., Peterkova R., Kangas A., Yu B., Lesot H., Peterka M., Jernvall J., Martin G. (2006). Sprouty genes control diastema tooth development via bidirectional antagonism of epithelial–mesenchymal fgf signaling. Dev. Cell.

[bib30] Kriangkrai R., Chareonvit S., Yahagi K., Fujiwara M., Eto K., Iseki S. (2006). Study of Pax6 mutant rat revealed the association between upper incisor formation and midface formation. Dev. Dyn..

[bib31] Laurikkala J., Mikkola M., Mustonen T., Aberg T., Koppinen P., Pispa J., Nieminen P., Galceran J., Grosschedl R., Thesleff I. (2001). TNF signaling via the ligand–receptor pair ectodysplasin and edar controls the function of epithelial signaling centers and is regulated by Wnt and activin during tooth organogenesis. Dev. Biol..

[bib32] Liodis P., Denaxa M., Grigoriou M., Akufo-Addo C., Yanagawa Y., Pachnis P. (2007). Lhx6 activity is required for the normal migration and specification of cortical interneuron subtypes. J. Neurosci..

[bib33] MacKenzie A., Ferguson M.W., Sharpe P.T. (1992). Expression patterns of the homeobox gene, *Hox-8*, in the mouse embryo suggest a role in specifying tooth initiation and shape. Development.

[bib34] Mandler M., Neubüser A. (2001). FGF signaling is necessary for the specification of the odontogenic mesenchyme. Dev. Biol..

[bib35] Marigo V., Johnson R.L., Vortkamp A., Tabin C.J. (1996). Sonic hedgehog differentially regulates expression of GLI and GLI3 during limb development. Dev. Biol..

[bib36] Matsumoto K., Tanaka T., Furuyama T., Kashihara Y., Mori T., Ishii N., Kitanaka J., Takemura M., Tohyama M., Wanaka A. (1996). L3, a novel murine LIM-homeodomain transcription factor expressed in the ventral telencephalon and the mesenchyme surrounding the oral cavity. Neurosci. Lett..

[bib37] Matsumoto K., Tanaka T., Furuyama T., Kashihara Y., Ishii N., Tohyama M., Kitanaka J., Takemura M., Mori T., Wanaka A. (1996). Differential expression of LIM-homeodomain genes in the embryonic murine brain. Neurosci. Lett..

[bib38] McLeod M.J. (1980). Differential staining of cartilage and bone in whole mouse fetuses by alcian blue and alizarin red S. Teratology.

[bib39] Mikkola M.L., Thesleff I. (2003). Ectodysplasin signaling in development. Cytokine Growth Factor Rev..

[bib40] Mina M., Kollar E.J. (1987). The induction of odontogenesis in non-dental mesenchyme combined with early murine mandibular arch epithelium. Arch. Oral Biol..

[bib41] Mitsiadis T.A., Angeli I., James C., Lendahl U., Sharpe P.T. (2003). Role of Islet1 in the patterning of murine dentition. Development.

[bib43] Mustonen T., Pispa J., Mikkola M.L., Pummila M., Kangas A.T., Pakkasjärvi L., Jaatinen R., Thesleff I. (2003). Stimulation of ectodermal organ development by ectodysplasin-A1. Dev. Biol..

[bib44] Ogawa T., Kapadia H., Feng J.Q., Raghow R., Peters H., D Souza R.N. (2006). Functional consequences of interactions between *Pax9* and *Msx1* genes in normal and abnormal tooth development. J. Biol. Chem..

[bib46] Pearse R.V., Vogan K.J., Tabin C.J. (2001). Ptc1 and Ptc2 transcripts provide distinct readouts of Hedgehog signaling activity during chick embryogenesis. Dev. Biol..

[bib47] Persson M., Stamataki D., te Welscher P., Andersson E., Böse J., Rüther U., Ericson J., James B. (2002). Dorsal-ventral patterning of the spinal cord requires Gli3 transcriptional repressor activity. Genes Dev..

[bib48] Peterková R., Peterka M., Viriot L., Lesot H. (1993). Multiple developmental origin of the upper incisor in mouse: histological and computer assisted 3-D-reconstruction studies. Int. J. Dev. Biol..

[bib49] Peterková R., Peterka M., Vonesch J.L., Ruch J.V. (2002). Development of the vestigial tooth primordia as part of mouse odontogenesis. Connect. Tissue Res..

[bib50] Peters H., Balling R. (1999). Teeth. Where and how to make them. Trends Genet..

[bib52] Quinn J.C., West J.D., Kaufman M.H. (1997). Genetic background effects on dental and other craniofacial abnormalities in homozygous small eye (Pax6Sey/Pax6Sey) mice. Anat. Embryol..

[bib53] Riddle R.D., Johnson R.L., Laufer E., Tabin C. (1993). Sonic hedgehog mediates the polarizing activity of the ZPA. Cell.

[bib54] Rivera-Pérez J.A., Mallo M., Gendron-Maguire M., Gridley T., Behringer R.R. (1995). Goosecoid is not an essential component of the mouse gastrula organizer but is required for craniofacial and rib development. Development.

[bib55] Sagasti A., Hobert O., Troemel E.R., Ruvkun G., Bargmann C.I. (1999). Alternative olfactory neuron fates are specified by the LIM homeobox gene *lim-4*. Genes Dev..

[bib57] Satokata I., Ma L., Ohshima H., Bei M., Woo I., Nishizawa K., Maeda T., Takano Y., Uchiyama M., Heaney S., Peters H., Tang Z., Maxson R., Maas R. (2000). Msx2 deficiency in mice causes pleiotropic defects in bone growth and ectodermal organ formation. Nat. Genet..

[bib58] Schaeren-Wiemers N., Gerfin-Mose A. (1993). A single protocol to detect transcripts of various types and expression levels in neural tissue and cultured cells: in situ hybridization using digoxigenin-labelled cRNA probes. Histochemistry.

[bib59] Schmeichel K.L., Beckerle M.C. (1994). The LIM domain is a modular protein-binding interface. Cell.

[bib60] Shibaguchi T., Kato J., Abe M., Tamamura Y., Tabata M.J., Liu J.-G., Iwamoto M., Wakisaka S., Wanaka A., Kurisu K. (2003). Expression and role of Lhx8 in murine tooth development. Arch. Histol. Cytol..

[bib62] Thomas B.L., Tucker A.S., Qui M., Ferguson C.A., Hardcastle Z., Rubenstein J.L., Sharpe P.T. (1997). Role of *Dlx-1* and *Dlx-2* genes in patterning of the murine dentition. Development.

[bib63] Tissier-Seta J.P., Mucchielli M.L., Mark M., Mattei M.G., Goridis C., Brunet J.F. (1995). Barx1, a new mouse homeodomain transcription factor expressed in cranio-facial ectomesenchyme and the stomach. Mech. Dev..

[bib64] Trumpp A., Depew M.J., Rubenstein J.L., Bishop J.M., Martin G.R. (1999). Cre-mediated gene inactivation demonstrates that FGF8 is required for cell survival and patterning of the first branchial arch. Genes Dev..

[bib65] Tucker A.S., Sharpe P.T. (2004). The cutting-edge of mammalian development; how the embryo makes teeth. Nat. Rev., Genet..

[bib66] Tucker A.S., Matthews K.L., Sharpe P.T. (1998). Transformation of tooth type induced by inhibition of BMP signaling. Science.

[bib67] Tucker A.S., Yamada G., Grigoriou M., Pachnis V., Sharpe P.T. (1999). Fgf-8 determines rostral-caudal polarity in the first branchial arch. Development.

[bib69] Tucker A.S., Headon D.J., Courtney J.-M., Overbeek P., Sharpe P.T. (2004). The activation level of the TNF family receptor, Edar, determines cusp number and tooth number during tooth development. Dev. Biol..

[bib70] Vainio S., Karavanova I., Jowett A., Thesleff I. (1993). Identification of BMP-4 as a signal mediating secondary induction between epithelial and mesenchymal tissues during early tooth development. Cell.

[bib71] Washbourne B.J., Cox T.C. (2006). Expression profiles of cIRF6, cLHX6 and cLHX7 in the facial primordia suggest specific roles during primary palatogenesis. BMC Dev. Biol..

[bib72] Yamada G., Mansouri A., Torres M., Stuart E.T., Blum M., Schultz M., De Robertis E.M., Gruss P. (1995). Targeted mutation of the murine goosecoid gene results in craniofacial defects and neonatal death. Development.

[bib73] Zhang Y., Zhang Z., Zhao X., Yu X., Hu Y., Geronimo B., Fromm S.H., Chen Y.P. (2000). A new function of BMP4: dual role for BMP4 in regulation of Sonic hedgehog expression in the mouse tooth germ. Development.

[bib74] Zhao Y., Guo Y.J., Tomac A.C., Taylor N.R., Grinberg A., Lee E.J., Huang S., Westphal H. (1999). Isolated cleft palate in mice with a targeted mutation of the LIM homeobox gene *lhx8*. Proc. Natl. Acad. Sci. U. S. A..

